# Social Enterprise Model (SEM) for private sector tuberculosis screening and care in Bangladesh

**DOI:** 10.1371/journal.pone.0241437

**Published:** 2020-11-23

**Authors:** Sayera Banu, Farhana Haque, Shahriar Ahmed, Sonia Sultana, Md. Mahfuzur Rahman, Razia Khatun, Kishor Kumar Paul, Senjuti Kabir, S. M. Mazidur Rahman, Rupali Sisir Banu, Md. Shamiul Islam, Allen G. Ross, John D. Clemens, Robert Stevens, Jacob Creswell

**Affiliations:** 1 icddr,b (International Centre for Diarrhoeal Diseases Research Bangladesh), Dhaka, Bangladesh; 2 National Tuberculosis Control Program (NTP), Ministry of Health and Welfare, Dhaka, Bangladesh; 3 Stop TB Partnership, Geneva, Switzerland; The University of Georgia, UNITED STATES

## Abstract

**Background:**

In Bangladesh, about 80% of healthcare is provided by the private sector. Although free diagnosis and care is offered in the public sector, only half of the estimated number of people with tuberculosis are diagnosed, treated, and notified to the national program. Private sector engagement strategies often have been small scale and time limited. We evaluated a Social Enterprise Model combining external funding and income generation at three tuberculosis screening centres across the Dhaka Metropolitan Area for diagnosing and treating tuberculosis.

**Methods and findings:**

The model established three tuberculosis screening centres across Dhaka Metropolitan Area that carried the icddr,b brand and offered free Xpert MTB/RIF tests to patients visiting the screening centres for subsidized, digital chest radiographs from April 2014 to December 2017. A network of private and public health care providers, and community recommendation was formed for patient referral. No financial incentives were offered to physicians for referrals. Revenues from radiography were used to support screening centres’ operation. Tuberculosis patients could choose to receive treatment from the private or public sector. Between 2014 and 2017, 1,032 private facilities networked with 8,466 private providers were mapped within the Dhaka Metropolitan Area. 64, 031 patients with TB symptoms were referred by the private providers, public sector and community residents to the three screening centres with 80% coming from private providers. 4,270 private providers made at least one referral. Overall, 10,288 pulmonary and extra-pulmonary tuberculosis cases were detected and 7,695 were bacteriologically positive by Xpert, corresponding to 28% of the total notifications in Dhaka Metropolitan Area.

**Conclusion:**

The model established a network of private providers who referred individuals with presumptive tuberculosis without financial incentives to icddr,b’s screening centres, facilitating a quarter of total tuberculosis notifications in Dhaka Metropolitan Area. Scaling up this approach may enhance national and international tuberculosis response.

## Introduction

Globally, national tuberculosis programs (NTPs) detect, treat and report seven of every ten people who are estimated to develop tuberculosis (TB) each year [1]. Annually, this gap translates to about 3 million people with TB who are missed, the majority of them residing in Asia where poor linkages of NTPs with the private sector is thought to be one of the biggest shortfalls [1–3]. In most Asian countries, the private sector is the first point of entry in health system [4, 5], and private health care providers (PPs) treat many people with TB. However, these individuals may be getting sub-optimal treatment due to delay in diagnosis, prescription of improper treatment regimens, deficient treatment support and monitoring, and poor quality drugs [2, 6–8]. Private sector engagement strategies are recognized as a critical aspect of the TB response [9]. Early approaches primarily focused on small groups of PP trained to refer people with TB symptoms to public facilities for diagnosis and/or treatment [2, 8]. While these approaches often showed small scale success, they did not engage deeply in the complicated web of factors driving private care and generally remained pilots. Recently, more nuanced approaches to engage the private sector have been initiated by non-public sector agents and have shown impressive results. These modern versions of private sector engagement take much more active roles in the process, are initiated by non-public sector agents, and have often produced impressive results by vastly increasing the numbers of people tested and leading to improved numbers of TB cases detection, treatment and reporting to NTPs. Yet sustainability of these approaches are always an issue as they require major knowledge and behaviour change as well as enforcement which often is not possible due to lack of funding and governance [8, 10–13]. The delivery of any health services including TB services, public or private, requires funding. This funding mainly comes from three sources: public sector commitments, patients’ out of pocket expenditure, and donor financing.

Despite a successful multi-sectoral TB response [14], a free, smear microscopy-based laboratory system, high treatment success, and improving case detection, Bangladesh still misses approximately 40% of the people who develop TB [2–5]. Although smear microscopy is inexpensive [15], its sensitivity is poor and quality is variable [16], thus negative results are often ignored by PPs [10]. As a result, most private sector clinicians prefer chest radiographs (CXR) or serological assays for TB diagnosis [10] which have their own limitations. CXR alone often leads to erroneous TB diagnosis as it is non-specific [17], and serological assays are unable to distinguish between infection and active TB disease [18]. Rapid molecular tests such as the GeneXpert MTB/RIF assay (Xpert) with higher sensitivity to detect TB as well as rifampicin resistance, is well positioned to be used as a point-of-care test [19]. However, Xpert currently has a price of US$ 9·98 for public-sector users [20], for which private sector patients are not eligible, passing extra costs to patients and rendering it unaffordable to most Bangladeshis [21] whose average monthly income is US$ 158 [22]. Referral systems for free Xpert testing may be an attractive way to link public- and private-sector providers. However, efforts to engage PPs are likely to fail if such approaches disrupt the intricate web of commercial incentives within private healthcare markets and hamper the generation of referral fees [23].

The clear need for sustained private sector engagement in Bangladesh and the opportunity to introduce Xpert on a large-scale prompted the design of an intervention linking individuals seeking care from PPs to free Xpert testing after the use of paid CXR services at three TB screening centres (SCs) across Dhaka employing a social enterprise model (SEM). A Social Enterprise [24] is defined as *“an organization or venture that advances its primary social or environmental mission using business methods*.*”* This usually includes some degree of cost recovery for the provision of socially valuable goods or services to a target population. Like any business, Social Enterprises must generate enough revenue to cover costs in order to continue operating, but unlike a traditional for-profit corporation, the surplus generated is used to either cross-subsidize socially valuable services that may generate a loss, or to expand the enterprise. Social Enterprises often use the concept of a “double bottom line”, where documented success is determined by both financial outcome and social impact. SEM have several advantages over traditional grant-based operations, including: sustainability *(*charging for goods/services allows the institution to develop an independent and sustainable internal revenue stream that can continue in perpetuity, rather than relying on availability of external grants), *accountability (*since patients have to use their own money to purchase goods/services, they will only do so if what is provided creates real value for them), and *scalability* (the surplus generated from a successful social enterprise can be used to expand the enterprise to different areas and populations). Also, SEMs also face a different set of constraints and issues, including: *complexity* (Social Enterprises must face the more complex management and accounting issues of managing revenues as well as costs, while providing and documenting a social benefit. Tax and regulatory factors may also be more complex, and will likely vary from country to country) and *equity* (since revenues must be generated to sustain operations, ability to pay becomes a criterion for receiving services—not need alone. Revenues from higher-income populations can be used to cross-subsidize lower-income ones, and such scenarios would require a rigorous method to means-test to limit the impact of non-revenue-generating services).

## Methods

### Ethical implications

The protocol (#PR-13003) and intervention was reviewed and approved by the Research Review Committee and the Ethical Review Committee at icddr,b. All data were collected after taking written informed consent from all the participants using icddr,b IRB approved consent form.

### Intervention setting

The intervention was conducted in Dhaka Metropolitan Area (DMA) with 132 basic management units (BMUs) offering TB services to an estimated 19 million population in 2018 [25]. Of these BMUs, 17 were operated in tertiary healthcare facilities and 115 at non-government organization (NGOs) premises. The BMUs were linked with NTP system and had overall responsibility for diagnosing, reporting and treating TB cases according to WHO guidelines following well-structured protocols. BMUs reported standard detection and outcome data to the NTP on a quarterly basis. The BMUs were funded by both national government and international donors through the NTP. DMA is one of the most densely-populated megacities in the world with a population of 8.5 million and more than one third (3.4 million) of its population living in slums [25] (details are provided in S1 File).

### Intervention

In private sector, physicians mostly depend on X-ray and smear microscopy findings to diagnose TB and mainly refer patients to privately run diagnostic centres. The SEM was developed by icddr,b (the International Centre for Diarrhoeal Diseases Research, Bangladesh), a leading health research institution globally, and carried the icddr,b brand. The SEM developed a referral network of public and private healthcare facilities who directed individuals with presumptive TB to three SCs where subsidized, digital CXR and free Xpert testing were available, providing an alternative to microscopy or CXR alone and shift the current dependency on AFB/culture test to more bacteriological evidence-based practice. The SEM became operational from July 2014 and is still operating. In this paper we present a performance evaluation of the SEM from July 2014 − December 2017.

**Fig 1:**
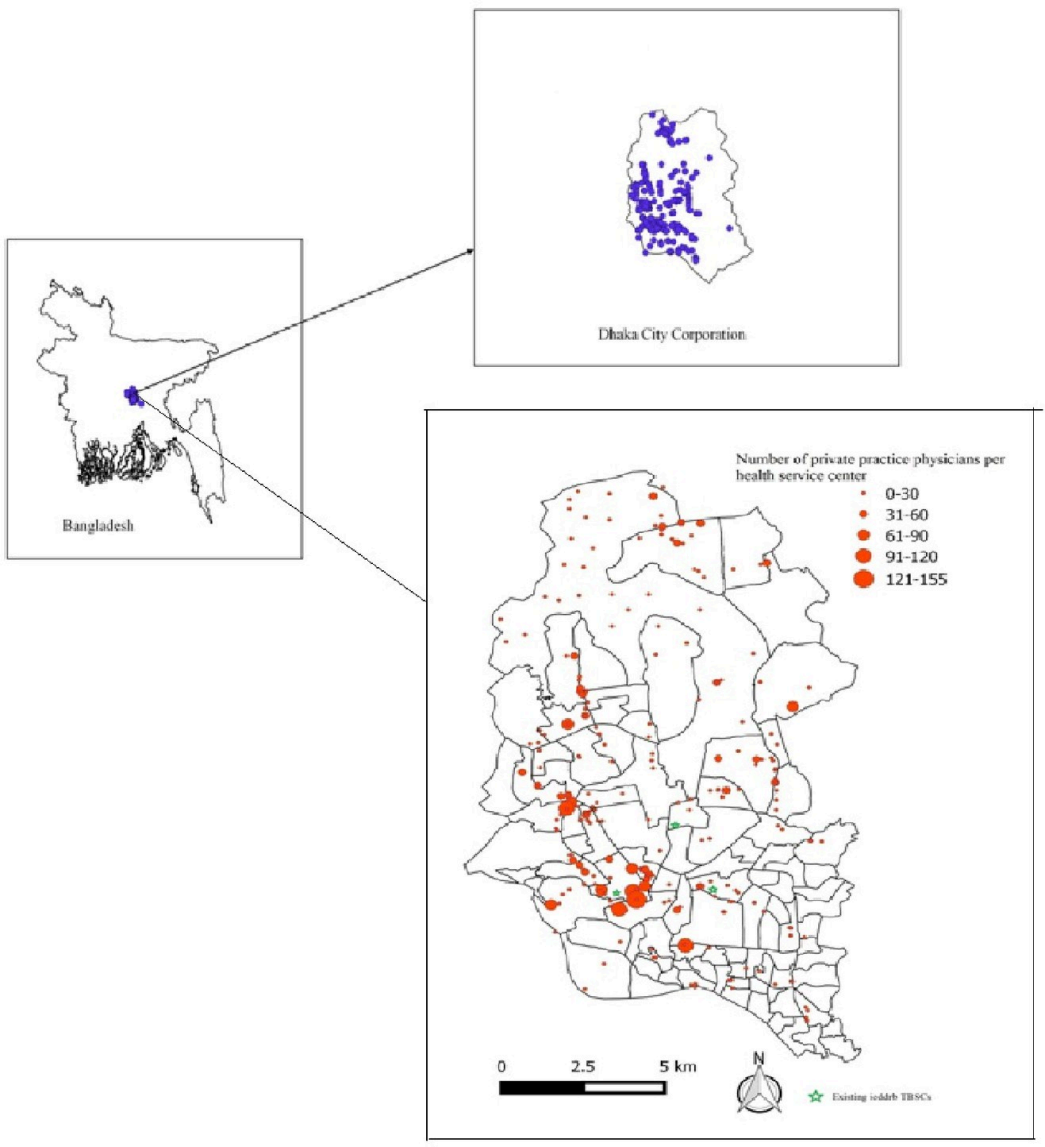
GIS mapped referral network of private healthcare facilities (upper right) and registered medical practitioners (lower right) within DMA.

The DMA was divided into four zones and over a three-month preparatory period, three field workers extensively visited each zone to identify and map all private healthcare facilities and providers including clinics, hospitals, diagnostic centres, pharmacies, individual and group practitioners' chambers offering TB referral diagnostic and/or treatment services. At each facility, the number of registered medical practitioners and GPS coordinates were collected (Fig 1).

SC locations were selected purposively from areas with high population density gradient (see S2 Fig), PP density, and access to public transport to increase coverage and accessibility. The SCs were equipped with Delft EZ DR X-ray systems and multiple four-module GeneXpert systems. In addition to the private sector referrals, the SCs encouraged referrals from the public sector for people with negative smear microscopy results, referrals from private pharmacies, and investigated household contacts of people identified with TB.

**Fig 2:**
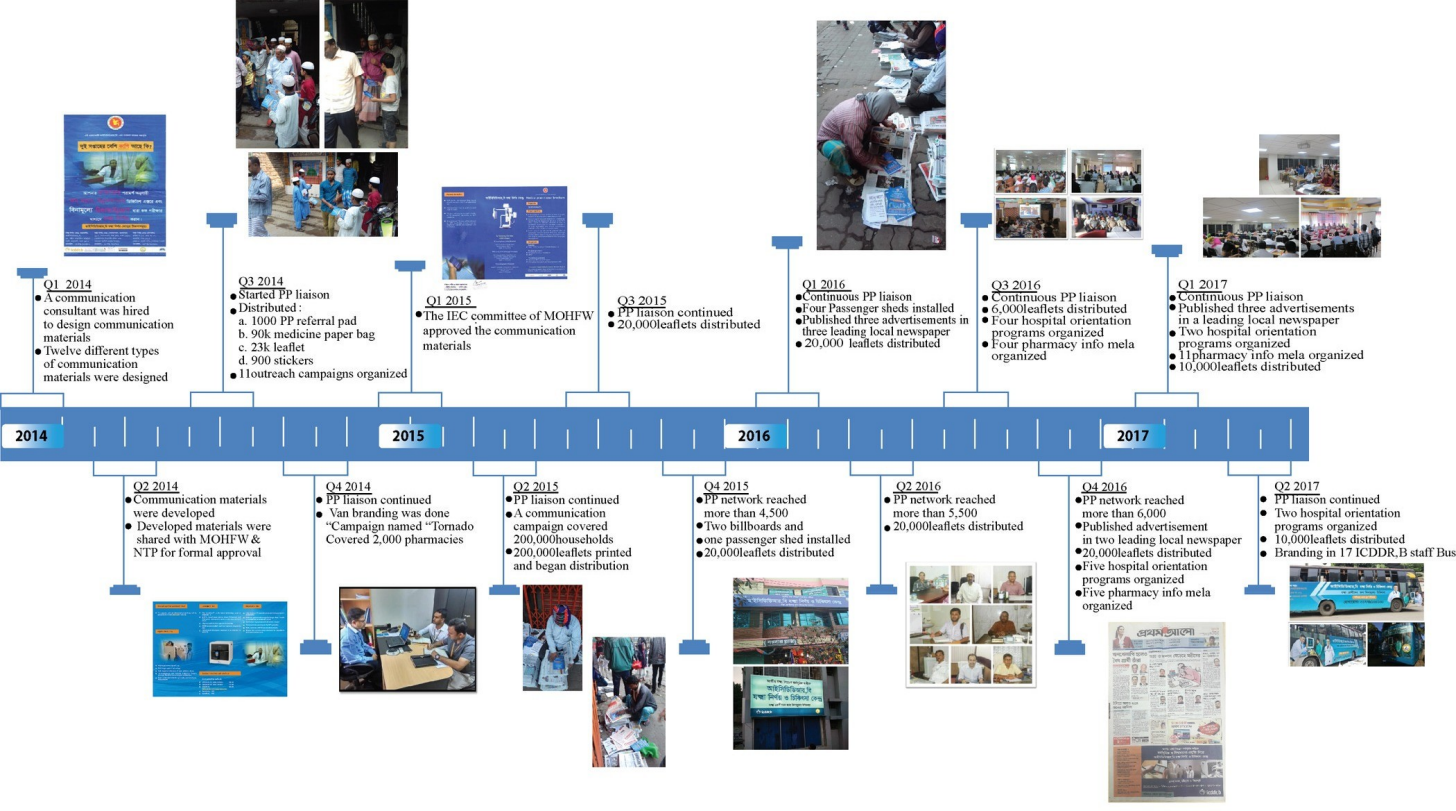
Timeline showing the strategies/activities for the development and reinforcement of private provider referral network.

Once mapped, field workers revisited each facility to network and orient PPs regarding SC services. Additional reinforcement through printed and other sensitization materials including short message service (SMS) were provided to the PPs to remind them about SC services during national and international events over the course of the intervention (Fig 2).

Pharmacies near each of the SC were visited by the liaising team in a non-systematic manner to encourage the pharmacies to make referrals. A total of 610 pharmacies were invited to participate in orientation trainings organized intermittently throughout the intervention period to sensitize pharmacists and drug sellers about TB management and referral options. Of them, 423 pharmacies took part in training.

All 132 BMUs were also eligible for referring patients with negative smear results for Xpert testing. SC staff visited the BMUs to reinforce engagement and promote referrals at regular intervals. From June 2014, household contact investigation was conducted for any person diagnosed with bacteriologically positive (B+) TB at the SCs. Contacts with TB symptoms were identified using telephone surveys and were invited to the SCs for further evaluation. SC health workers visited the household if symptomatic contacts were unable to visit a SC for sample collection and testing.

An awareness campaign was designed to promote the SCs’ TB testing services. Promotion was done through outreach campaigns, newspaper advertisements, stickers and referral pad distribution, public announcements, three large billboard advertisements on major roads, social media (Facebook), and distribution of posters/leaflets at public gathering points including mosques, schools, bus terminals etc.

We used a decentralized implementation of Xpert testing and reporting using community health workers with no prior laboratory experience for the SEM intervention. We hired people with 12 years of completed school education with previous work experience of working in any kind of laboratory facilities or sample handling to run the SCs lab facilities. For networking with physicians and other health practitioners, we hired people with graduate/masters level education with experience of working in health sectors in any capacity. We also hired a few medical graduates to build up the PP network. All staff hired were then trained on classroom-based theory, technique and computer training by the icddr,b project team and senior lab technicians prior to the start of the project. The classroom training was complemented by hands on training with senior lab technicians in the icddr,b lab for conducting the Xpert tests and communication with private providers. Finally, in service training and supervision was provided for all trained staff. This supervision continued until senior lab technicians and project staff became comfortable with the performance of the newly trained community health workers and PP liaison workers.

### Testing algorithm

**Fig 3:**
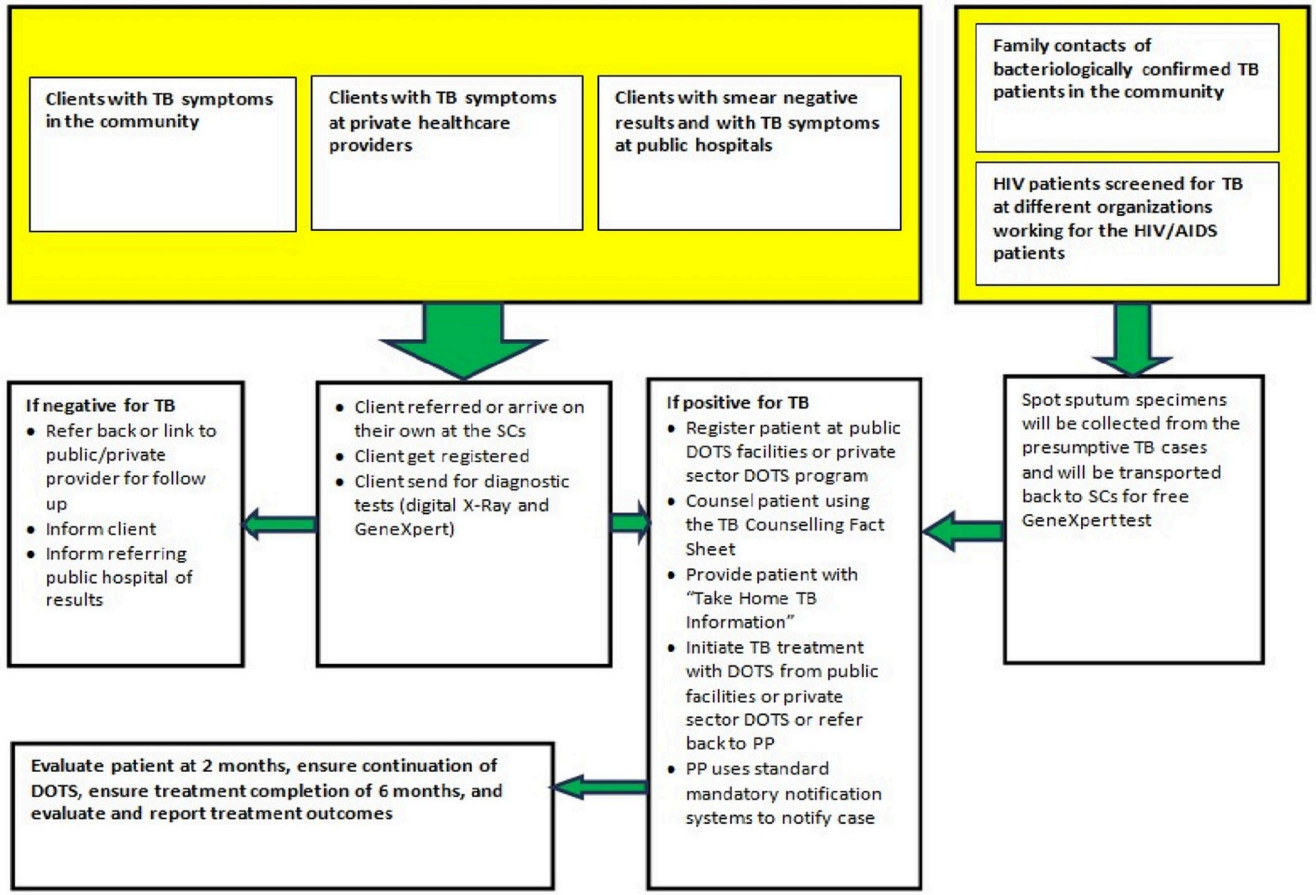
Algorithm of intervention services at the icddr,b SEM screening centres (2014−2017).

Staff at the SCs used a mobile phone application to collect demographic, clinical and exposure information from all presumptive cases before beginning diagnostic evaluation. Individuals arriving at a SC with a cough of two weeks or more or other symptoms suggestive of TB (fever, night sweats, and/or weight loss), were defined as presumptive pulmonary TB cases. Also, the SCs offered service to presumptive extra-pulmonary TB cases referred by a healthcare provider. Eligible individuals were offered a subsidized CXR (~4 USD), which was immediately analyzed using CAD4TB software and read by a radiologist following established procedures that have been described elsewhere [26]. These individuals were then offered a free Xpert test for bacteriological confirmation. If the Xpert test failed (invalid, error, or no result), a second test was performed to obtain a valid outcome if enough sample remained. Treatment initiation and outcomes were recorded for all people diagnosed to have TB. People with positive Xpert results were counseled and referred for free treatment at the BMU nearest to their residence following the NTP guidelines. Those wanting to remain with their referring physicians were referred back for TB treatment with drugs purchased from pharmacies. Patients with negative test results were followed up over phone to detect clinically diagnosed TB cases. The SC staff followed-up these patients (both B+ and clinically diagnosed) through regular phone calls and tracked and reported their outcomes to the NTP. Individuals with rifampicin-resistant TB results were referred to the National Institute of Diseases of the Chest and Hospital (NIDCH) for additional testing, clinical evaluation and second-line treatment initiation (Fig 3). All three SCs followed the same procedures for TB screening.

### Revenue generation

The fee charged for CXR in the SCs (<4 USD) was on the lower end of the digital CXR market price in the DMA. All revenues generated from the CXR services were reinvested for SCs staff salary, office and laboratory supplies procurement, and maintenance costs. The SCs also provided a service denominated as “commercial service” by serving as sample collection sites for paid general laboratory testing services (AFB/Culture/Xpert/Extra-pulmonary samples) from the commercial diagnostic laboratories at icddr,b. In return, the SCs received fifteen percent of the revenue. Free CXR was available to select individuals when their PPs requested for fee waiver.

### Data collection procedure and analysis

All data were collected by trained data collectors using a custom-built mHealth application or software installed on Java platform mobile phones and securely transmitted to a mySQL database connected to a medical record system (OpenMRS). The OpenMRS applied multi layers of security to provide privacy, and confidentiality of data throughout the data life cycle. These layers were based on organizational and government national policy on data security. The technology used in data collection, management, storage, and use; as well as user behavior were carefully analyzed to identify and protect potential vulnerabilities. To protect the sensitive health data our mobile apps developers built a system that had a secure back-end database and ensured minimal or no personal health information on the device and the hardware, software, and communication channels between the device and other systems were secured.

A structured questionnaire was used to collect information on socio-demographic characteristics, referral information, clinical sign/symptoms, previous history of TB, and other illness etc. from presumptive patients. Data was collected regarding the number of people screened for TB symptoms, presumptive TB individuals identified, CXR and Xpert tests performed, type of TB detected, and treatment outcomes. We also collected data on fixed costs (rent, insurance, financing costs, maintenance and repairs, utilities, salaries, advertising, administrative costs, depreciation of fixed assets) as well as variable (running) costs for the SCs. De-identified data were extracted from the project database and analyzed using STATA version 13. Descriptive statistics including Chi-square test and/or Fisher exact tests were generated to compare referral data between categories of PPs. NTP notification data was used to measure the contribution of SEM on case notification.

## Results

During the mapping exercise, 1,032 private healthcare facilities were identified (Annex 2: e Fig 1). All facilities were visited regularly, and a network was established with 8,466 PPs (52% of the estimated PPs in the DMA). The PPs included 1,517 chest physicians, 2,511 general practitioners, and 4,438 internists and other specialists.

During the evaluation, 64,031 people with TB symptoms including 2,839 children under the age of 15 years attended the SCs. Among them, 57,580 (90%) sought TB screening services, while 6,451 sought other commercial services. Overall, 80% (n = 51,129) of people tested at the SCs were referred by PPs. Walk-ins or self-referrals, referrals from BMUs, pharmacies and other informal providers, and contact investigation contributed to 12% (n = 7,424), 6% (n = 3,859), 1% (n = 959) and 1% (n = 660) respectively (Table 1).

**Table 1:** Summary results of TB screening by strategy at the icddr,b SEM screening centres (2014−2017).

Factors						
	Total	Private Providers	DOTS/ BMUs	Walk-ins	Contact investigation	Pharmacies
	N	n (%)	n (%)	n (%)	n (%)	n (%)
**Attendees served at the SCs**	**64031**	**51129 (80)**	**3859 (6)**	**7424 (12)**	**660 (1)**	**959 (1)**
Adults (≥15)	61192	48893 (80)	3687 (6)	7104 (12)	598 (1)	910 (1)
Children (<15)	2839	2236 (79)	172 (6)	320 (11)	62 (2)	49 (2)
**Screening service**						
Attendees came for TB screening	**57580**	**45778 (80)**	**3421 (6)**	**6856 (12)**	**605 (1)**	**920 (2)**
Presumptive cases received a CXR	54125	42465 (78)	3388 (6)	6764 (12)	571 (1)	937 (2)
Presumptive cases provided sputum	53730	42494 (79)	3250 (6)	6537 (12)	567 (1)	882 (2)
First time Failed Xpert tests	1350	1064 (79)	110 (8)	146 (11)	12 (1)	18 (1)
**TB patients diagnosed by Xpert test**	**7695**	**6277 (82)**	**518 (7)**	**737 (10)**	**69 (1)**	**94 (1)**
RIF^a^ Sensitive	7247	5928 (82)	481 (7)	682 (9)	67 (1)	89 (1)
RIF Indeterminate	109	82 (75)	10 (9)	15 (14)	1 (1)	1 (1)
RIF Resistant	339	267 (79)	27 (8)	40 (12)	1 (0)	4 (1)
**TB patients diagnosed clinically**	1455	1195 (82)	95 (7)	137 (9)	11 (1)	17 (1)
Commercial service						
**TB patients detected by Xpert test**	183	183 (100)	0 (0)	0 (0)	0 (0)	0 (0)
RIF Sensitive	170	170 (100)	0 (0)	0 (0)	0 (0)	0 (0)
RIF Indeterminate	3	3 (100)	0 (0)	0 (0)	0 (0)	0 (0)
RIF Resistant	10	10 (100)	0 (0)	0 (0)	0 (0)	0 (0)
**TB patients detected by AFB^b^ & Culture**	188	150 (80)	10 (5)	23 (12)	2 (1)	3 (2)
**EP-TB^c^ patients detected**	695	603 (87)	43 (6)	42 (6)	3 (0)	4 (1)
**TB patients diagnosed clinically**	72	72 (100)	0 (0)	0 (0)	0 (0)	0 (0)
**Total TB cases (all forms) identified**	**10288**	**8480 (82)**	**666 (6)**	**939 (9)**	**85 (1)**	**118 (1)**
Adults (≥15)	9982	8217 (82)	648 (6)	917 (9)	84 (1)	116 (1)
Children (<15)	306	263 (86)	18 (6)	22 (7)	1 (0)	2 (1)
Treatment status						
Patients died before treatment initiation	134	118 (88)	6 (4)	8 (6)	0 (0)	2 (1)
**Patients (all forms) started First-line TX^d^**	**8077**	**6711 (83)**	**480 (6)**	**736 (9)**	**64 (1)**	**86 (1)**
DOTS facilities	7039	5826 (83)	426 (6)	654 (9)	54 (1)	79 (1)
Private sector	1038	884 (85)	54 (5)	83 (8)	10 (1)	7 (1)
First line pre-treatment loss to follow up	1728	1374 (80)	153 (9)	155 (9)	20 (1)	26 (2)
**RR^e^ Patients started second-line TX**	254	206 (81)	21 (8)	26 (10)	0 (0)	1 (0)
RR Patients started first-line TX	7	4 (57)	1 (14)	2 (29)	0 (0)	0 (0)
Second line pre-treatment loss to follow up	88	67 (76)	5 (6)	12 (14)	1 (1)	3 (3)

^**a**^ RIF (resistance to Rifampicin),

^**b**^AFB (acid fast bacilli),

^**c**^EP-TB (extra-pulmonary TB),

^**d**^TX (treatment),

^**e**^RR (rifampicin-resistant)

Of the 8,466 PPs who agreed to participate in the referral network, 4,270 (50%) referred at least one person to SCs for testing, and 1,313 (16%) referred at least one person who was diagnosed with TB. A smaller number of PPs, 484 (6%) referred at least ten people. Among this group, 184 (38%) were internists or medicine specialists, and 175 (36%) were chest physicians. Overall, chest physicians referred 52% (n = 26,444) for testing and no differences in yield (TB among presumptive referrals) between provider types were found (Table 2).

**Table 2:** Referral characteristics of private providers networked within Dhaka metropolitan area.

Factors	Total N	Chest Physicians/Consultants	General Practitioners	Internists/ Specialists	Others	p-value
		n(%)	n(%)	n(%)	n(%)	
**Networked Providers^a^**	8466	1517 (18)	2511 (30)	2133 (25)	2305 (27)	
**Patients referred to SC^b^**	**51129**	**26444 (52)**	**3080 (6)**	**11021 (22)**	**10584 (21)**	
**Adults**	48894	25298 (52)	2948 (6)	10504 (21)	10144 (21)	p<0.001^f^
**Children**	2235	1146 (51)	132 (6)	517 (23)	440 (20)	p<0.05 ^g^
**Referred at least 1 patient**	4270	745 (17)	1410 (33)	1044 (24)	1071 (25)	p<0.05
**Referred at least 10 patient**	484	175 (36)	17 (4)	184 (38)	108 (22)	p<0.001
**Total TB Cases identified**	8480	5302 (63)	562 (7)	1515 (18)	1101 (13)	p<0.001
**Pulmonary B+ cases^c^**	6610	4247 (64)	346 (5)	1123 (17)	894 (14)	p<0.001
**Pulmonary B- cases** ^d^	1267	800 (63)	162 (13)	241 (19)	64 (5)	p<0.001
**EPTB**^e^ **cases**	603	255 (42)	54 (9)	151 (25)	143 (24)	p<0.001
**Referred ≥1 TB cases**	1313	358 (27)	269 (20)	391 (30)	295 (22)	p<0.05
**Referred ≥10 TB cases**	101	71 (70)	1 (1)	21 (21)	8 (8)	p<0.05

^a^ Exact enumeration of PPs in Dhaka is unavailable. According to Ministry of Health and Family Welfare (MOHFW) 2013, 62% of the 64,434 registered physicians were involved in the private sector. Among these, one-third (~16,000) of the total estimated private providers were expected to serve in the DMA [27].

^**b**^ SC (screening centre),

^**c**^ Pulmonary B+ cases (bacteriologically positive pulmonary TB cases).

^d ^Pulmonary B- cases (clinically diagnosed pulmonary TB cases)),

^**e**^ EPTB (extra-pulmonary TB).

^f^ p<0.001 in all comparisons between chest physician/consultant Vs all, internist/specialists Vs general practitioners, internist/specialists Vs others, other Vs general practitioners by chi square test or fisher exact test as appropriate

^g^ p<0.05 in all comparisons between chest physician/consultant Vs all, internist/specialists Vs general practitioners, internist/specialists Vs others, other Vs general practitioners by chi square test or fisher exact test as appropriate

Of the 132 BMUs, 126 (92%) made at least one referral of people with negative smear results. Among the 423 pharmacies in the network, 141 provided at least one referral and 48 referred at least one person with TB.

Among the people attending for TB screening, 54,125 (94%) received a CXR, and almost all paid (1%, n = 572 received a free CXR). Of those receiving a CXR, 53,730 (99%) provided sputum specimens for Xpert testing. Initially 1,350 (3%) of the Xpert tests failed, but most were retested, availing valid results for 53,726 (99.9%) individuals. Among these, 10,288 all forms TB cases were identified (9,150 from screening service and 1,138 from commercial service) including 306 children. From the screening service, 7,695 were B+ TB cases, while 1,455 were diagnosed clinically (Table 1). A total of 349 people had rifampicin resistance (RR). The majority of RR cases (n = 277, 79%) were referred by PPs, while the BMUs (n = 27, 8%), walk-ins (n = 40, 11%) and pharmacies (n = 4, 1%) contributed the remainder. Second line therapy was initiated in 254 (73%) RR cases.

**Fig 4:**
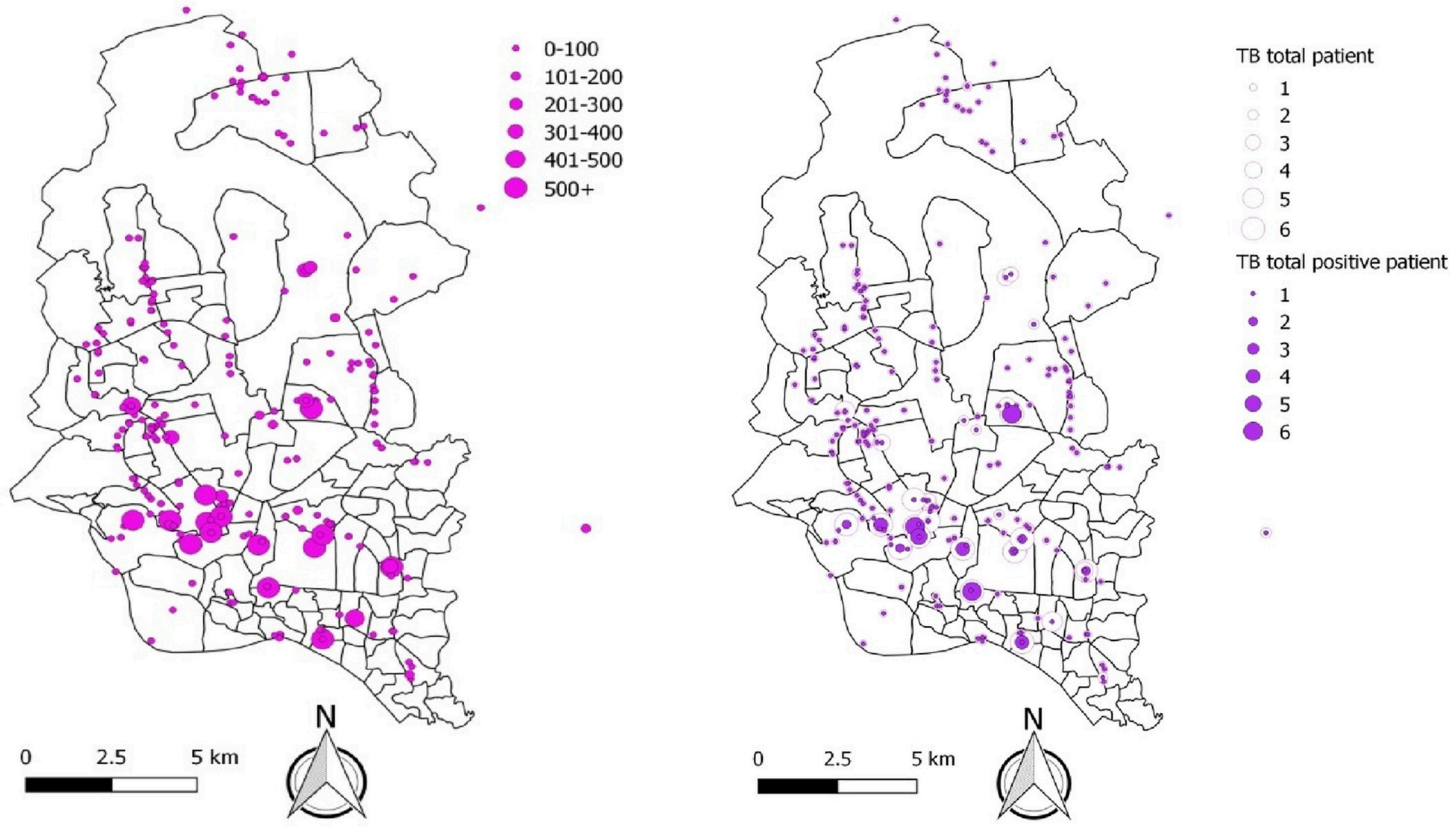
Distribution map of presumptive (left) and confirmed TB cases (right) identified by icddr,b SEM screening centres across DMA (2014–2017).

PP referrals accounted for 6,277 (82%) of B+ cases, while public sector referrals, walk-ins, contact investigation and pharmacies accounted for 518 (7%), 737 (10%), 69 (1%) and 94 (1%) respectively (Table 1). Among the PP referrals, 4,247 (64% of all B+ cases) were referred by chest disease consultants despite comprising only 18% of networked physicians (Table 2). Most of the people referred for TB screening (80%) and the people with B+ results (82%) came from the networked PPs (Fig 4).

Of the 7,695 B+ cases diagnosed at the SCs, contact tracing activities were conducted on 7,618 cases by telephone surveys and 4,952 contacts were screened. Telephone surveys recruited 660 contacts to the SCs, resulting in 71 B+ cases (11% of those tested and 1.4% of those screened) (Table 1). An additional 576 sputum samples (not presented in the table) were collected through home visits from contacts unable to visit the SCs, resulting in a total of 1,236 presumptive contacts. Four B+ individuals were identified from the contact samples collected from home.

Among the 8,077 individuals starting first-line treatment, the majority (7,039, 87%) chose to receive treatment at a BMU nearby their residence. However, 1,038 people (13%) chose to receive treatment from their private physicians and were supported by SC staff to collect official treatment outcomes (Table 1). The treatment success was slightly higher (75% vs 72%, p<0.05) at DOTS facilities than with PP while a slightly fewer patients died (7% vs. 9%) respectively p<0.05) (Table 3).

**Table 3:** Treatment outcomes of patients identified in screening centres by treatment location (2014–2017).

Treatment Outcomes	DOTS Facilities	Private Providers	p-value
	N = 7,039 (%)	N = 1,038 (%)	
**Treatment success^a^**	5,388 (77)	764 (74)	<0.05
**Treatment failure^b^**	76 (1)	12 (1)	0.749
**Died**	488 (7)	94 (9)	<0.05
**Loss to follow up**	1087 (15)	168 (16)	0.491

^**a**^ Tested smear-negative after completion of 6 months of treatment or completed 6 months of treatment but smear microscopy not done/test result could not be obtained;

^**b**^ Tested smear-positive after completion of 6 months of treatment.

In 2014, a total of 15,868 all form TB cases were notified to the NTP in DMA including 5,966 (38%) B+ cases. The three SCs detected 650 cases in total during the first 8 months of operation, which represented 11% of total B+ TB cases notified in the DMA in 2014. This proportion escalated to 37% of the total NTP notification by 2017 (3,079 of 8,332 cases). The number of drug resistant cases notified and treated also showed an increasing trend (Table 4).

**Table 4:** Contribution of icddr,b screening centres in notifying bacteriologically positive and drug resistant TB patients in Dhaka (2012 − 2017).

Year	All form TB cases notified in NTP^a^	All form TB cases notified in SCs^b^ n(%)	B (+) TB^c^ cases notified in NTP	B (+) TB cases notified by SCs n(%)	DR-TB^d^ cases notified in NTP	DR-TB cases notified by SCs n(%)
**2012**	14,056	NA^e^	6008	NA	505	NA
**2013**	14,469	NA	5750	NA	686	NA
**2014**	15,868	1080 (7)	5,966	650^f^(11)	946	40 (4)
**2015**	17654	944 (17)	6,517	1716 (26)	880	104 (12)
**2016**	18,408	33974 (18)	7,030	2250 (32)	918	119 (13)
**2017**	21,192	3729 (18)	8,332	3079 (37)	920	91 (10)

^**a**^ NTP (National Programme),

^**b**^ SCs (screening centres),

^**c**^ B (+) TB (Bacteriologically positive TB),

^**d**^ DR-TB (Drug resistant TB),

^**e**^ NA (Not applicable).

^**f**^ May—Dec 2014

The estimated PTB and EPTB case notification rate in DMA was between 61.66 to 65.67 per 100,000 population and 68.4 to 68.73 during 2015 to 2017 respectively. The calculated case notification rate of the SCs were similar to DMA with 61.98 to 65.88 per 100,000 population and 67.89 to 68.48 per100,000 population.

Monthly revenue generation started at US$ 2,464 and reached US$ 12,220 by the end of the intervention period. During the first 6 months, 9% of the total operational costs were recovered which increased to 37% by the end of the evaluation period. The majority of the revenue came from CXR fees (Table 5).

**Table 5:** Revenue (USD) generated by the icddr,b screening centres (2014−2017).

Year	Revenue from CXR^a^	Revenue from diagnostic services	Total revenue	Operational expenditure^b^	Proportion of expenditure recovered
**2014 (May-Dec)**	19,400	308	19,708	210,456	9%
**2015**	74,983	3,279	78,262	285,408	27%
**2016**	94,769	8,326	103,095	285,408	36%
**2017**	83,919	62,726	146,645	299,678	49%

^**a**^ CXR (Chest X-ray)

^**b**^ Operational expenses include rent, administrative and human resource costs to operate the Screening Centres, but do not include the cost of the diagnostic test procurement.

## Discussion

The results of our assessment show the considerable impact that private sector engagement using new molecular tests and digital imaging can have in Dhaka: three facilities detected 37% of B+ TB notifications in an Asian megacity. Moreover, unlike many recent private sector engagement initiatives, the proportion of B+ TB case detection was quite high [12, 28, 29]. We also found high numbers of people with drug-resistant TB from the private sector. Offering PPs free Xpert tests for their clients was an effective tool to link people who preferentially visit the private sector. Bangladesh has well-documented experiences of successful partnerships with NGOs in TB [30, 31] but published evidence is limited, and, none achieved such a large impact on notifications at a city level [31, 32].

In traditional private sector engagement strategies, PP refers patients to public sector sites for diagnosis [33]. Our model used private sector testing sites in the opposite way, by diagnosing TB from predominantly private and public sector referrals. Despite regular visit to the public BMUs to encourage referrals of people with smear negative results with suggestive clinical sign/symptoms, we were not able to test as many people as we had hoped, a clear missed opportunity gives the high yield of testing. However, the strategy was dependent on referrals being made by the BMUs.

A frequent critique of private sector engagement interventions is their lack of bacteriological confirmation for many individuals [34]. In our SEM, 75% of the cases identified were B+, compared to roughly 49% for the DMA (Table 4). The SEM improved confirmed case detection and ensured evidence-based management.

Our study is one of the first to map the contribution of different types of providers at a large scale. We found that significant proportion of people with TB were referred by a small number of specialized chest physicians. While these popular specialists are important to ensure effective private sector engagement, there are still considerable numbers of practitioners across Dhaka attending to people with TB. We covered an estimated 52% of Dhaka’s registered PPs. While we believe we engaged PPs treating the highest numbers of TB patients, we surely still missed others treating people with TB [35].

It was clear from the initial mapping exercises that many chest physicians are treating more people than are actually sent for Xpert testing. In Bangladesh, many PPs are hosted by commercial diagnostic centers with in-house CXR and laboratory facilities. This setup incentivizes hosted clinicians to refer their patients to in-house diagnostic facilities while receiving financial commissions. It is therefore unlikely the networked PPs referred all their patients with TB symptoms to SCs. Also, we believe the referring physicians likely only sent individuals they felt needed to receive advanced diagnostic testing and were willing to pay for CXR but cannot quantify this. These might be two of the major reasons behind not receiving patient referral from half of the networked PPs. Moreover, physicians referral of selective cases might be the reason behind detection of higher proportion of B+ TB cases in the SCs. A recent analysis from Indian drug sales data provides an excellent way of measuring private sector engagement coverage, and this approach could be used in future work [36]. Strategies that ensure notification of all people treated will help better understand the dynamics of the TB epidemic in Bangladesh.

This intervention identified 349 people with DR-TB and enrolled 254 of them on second-line treatment (8% of DR-TB enrolments in the DMA). In many countries, the majority of people with DR-TB have no known risk factors, meaning focusing testing on previously treated patients will fail to reach a large proportion of incident cases [37]. Universal drug-susceptibility testing is a national and global goal [4, 38], and Xpert testing of presumptive TB cases will increase the number of people identified with DR-TB and may help to bring down levels of drug resistance through early detection [39].

The use of CXR for diagnosing and triaging people who need expensive follow-on Xpert testing is gaining more and more interest. Using CXR to decide which individuals do not need Xpert testing could save funding. In a large evaluation of the artificial intelligence (AI) software to read CXR images we found that those human readers outperformed the machines. However, recent a study comparing new versions of the software demonstrated great improvement and better performance, including the ability to save large numbers of Xpert cartridges while missing few B+ TB cases and could be reconsidered in the future [40].

Low- and middle-income countries are increasingly trying to implement some method of contact investigation to improve case detection [41, 42]. This intervention is the first in Bangladesh to document contact tracing for people with TB from the private sector, and found similar results to other studies [41]. We also found some success among pharmacy referrals, but as other projects trying to link pharmacies have shown, great efforts are required to ensure pharmacy referrals [28, 43].

The SEM achieved 37% recovery of operational costs. While not enough to operate without additional funding, it begins to address the issue of sustainable healthcare financing [44]. Competing with PPs on referring patients for fee-based CXR resulted in fewer case referrals to the SCs then expected over the progression period of the intervention, and likely slowed the revenue growth.

Limitations of the intervention include lack of data on complete PP mapping and private sector drug sales. We were unable to track the cascade of care beginning with patient referrals to the SCs as we could not monitor how many PP referrals were made due to resource constraints. This model used a series of interventions conducted under programmatic conditions, not in a controlled environment. However, they give a sense of what results are possible in a ‘real-world’ setting for private sector engagement.

## Conclusions

The SEM engaged a large number of private care providers for client referral over the multiyear project period without financial incentives. The SEM improved TB and DR-TB cases notifications at a city level and helped to improve evidence-based TB management. This model, like others that use an interface agency to link the public and private sectors [12, 13], is a useful method of broader engagement of the private sector for TB care, especially in Asia. The revenue generation aspect of this model may provide a sustainable solution in the TB response.

## Supporting information

S1 File Study setting. (DOCX)

S1 Fig (TIF)

S2 Fig Population density gradient map of DMA. ^a^ This population contour map was developed using the methods explained in H. Khatun, N. Falgunee, M. J. R. Kutub (2015), Analyzing urban population density gradient of Dhaka Metropolitan Area using Geographic Information Systems (GIS) and Census Data, GEOGRAFIA OnlineTM Malaysian Journal of Society and Space 11 issue 13 (1–13).
(TIFF)

## Acronyms and abbreviations

B+: Bacteriologically positive
BMU/s: Basic management unit/s
CXR: Chest X-ray
DMA: Dhaka Metropolitan Area
DOTS: Directly Observed Treatment Short-course
DR-TB: Drug-resistant TB
DST: Drug-susceptibility test
GIS: Geographic information system
icddr,b: International Centre for Diarrhoeal Diseases Research, Bangladesh
OpenMRS: Medical record system
MOHFW: Ministry of Health and Family Welfare
NGO/s: Non-government organization/s
NIDCH: National Institute of Diseases of the Chest and Hospital
NTP/s: National TB Program/s
PP/s: Private provider/s
RR: Rifampicin resistance
SC/s: Screening Center/s
SEM: The Social Enterprise Model
SMS: Short message service
TB: Tuberculosis
Xpert: Xpert MTB/RIF assay
