# Supplementary material for: Social Enterprise Model (SEM) for private sector tuberculosis screening and care in Bangladesh
Source: PLoS One. 2020 Nov 23;15(11):e0241437. doi: 10.1371/journal.pone.0241437 (PMC7682881; doi:10.1371/journal.pone.0241437)
Supplement: S1 File — (DOCX) [file pone.0241437.s001.docx]

## S1 File: Study Setting

The DMA is divided into 92 municipal city wards and 855 *mahallas* (an optional and non-elective unit of a City corporation, considered as ‘neighborhoods’ of mega cities or town) and has 132 basic management units (BMUs) offering TB services. Of these BMUs, 17 were operated in tertiary healthcare facilities and 115 at non-government organization (NGOs) premises. BMUs reported standard detection and outcome data to the NTP on a quarterly basis. The private sector is a major provider of health care in Bangladesh, which include privately run hospitals/clinics general practitioners (GPs) chamber, consultants, charity/NGO run clinics, dispensaries, private laboratories and traditional healers. DMA has approximately 2000 private health care facilities and almost all of them have X-ray facilities. But except one hospital, Xpert testing is not available in any of them during the period of study evaluation. Also, Xpert use through the NTP or NGOs had been very limited in Bangladesh. In 2011, twelve Xpert systems were procured through USAID. Two of these systems have been set up in the National TB Reference Lab and Regional TB Reference Lab, but access to testing is limited to patients with suspected drug-resistant TB and smear-positive TB patients with a history of exposure to laboratory confirmed MDR TB cases. The remaining eight machines were deployed around the country following similar testing guidelines. Also, during the intervention period no new tests had been introduced nor any change was made in the treatment and diagnostic guideline by NTP.
