# Supplementary figures and images for: Social Enterprise Model (SEM) for private sector tuberculosis screening and care in Bangladesh
Source: PLoS One. 2020 Nov 23;15(11):e0241437. doi: 10.1371/journal.pone.0241437 (PMC7682881; doi:10.1371/journal.pone.0241437)

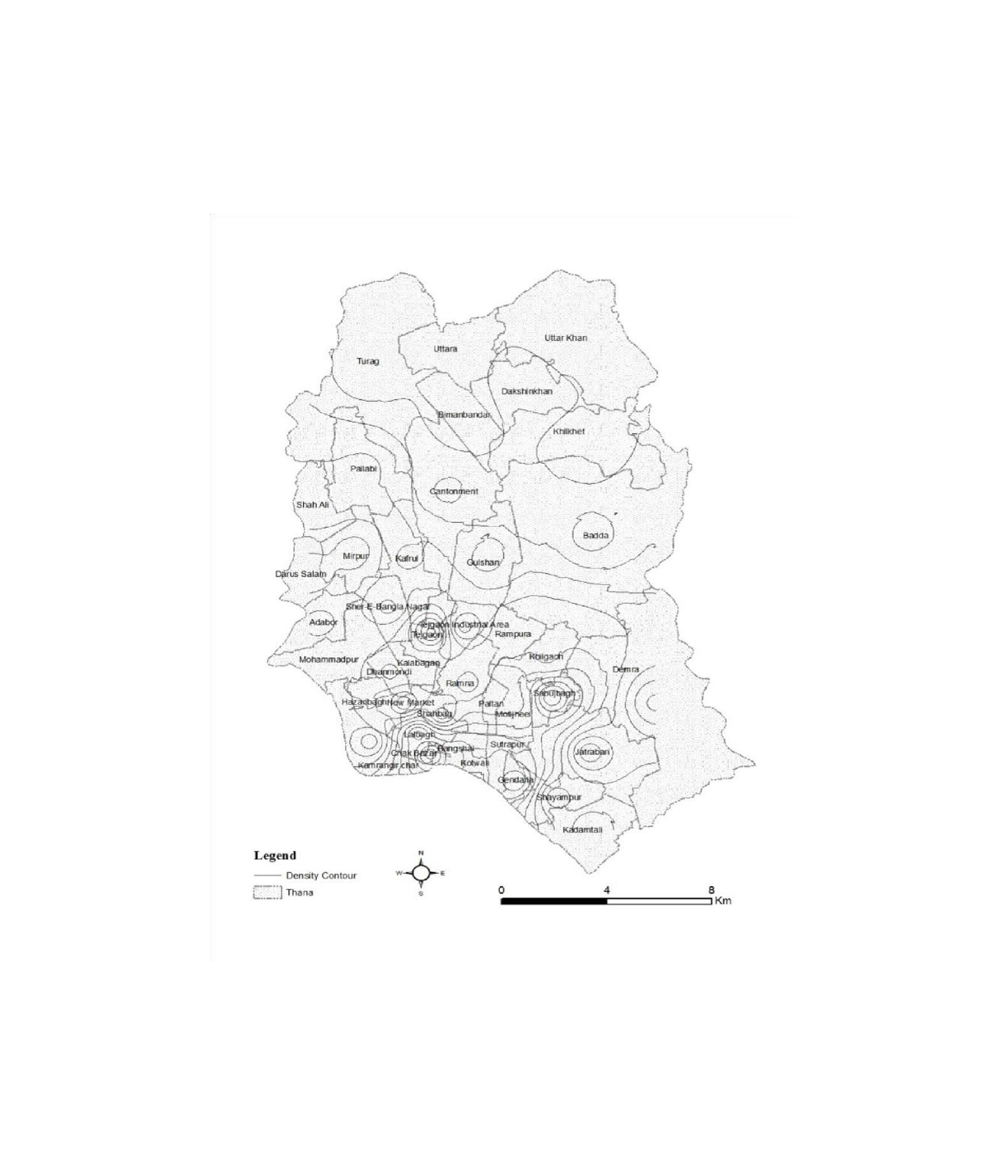

Supplement: S1 Fig — (TIF) [file pone.0241437.s002.tif]

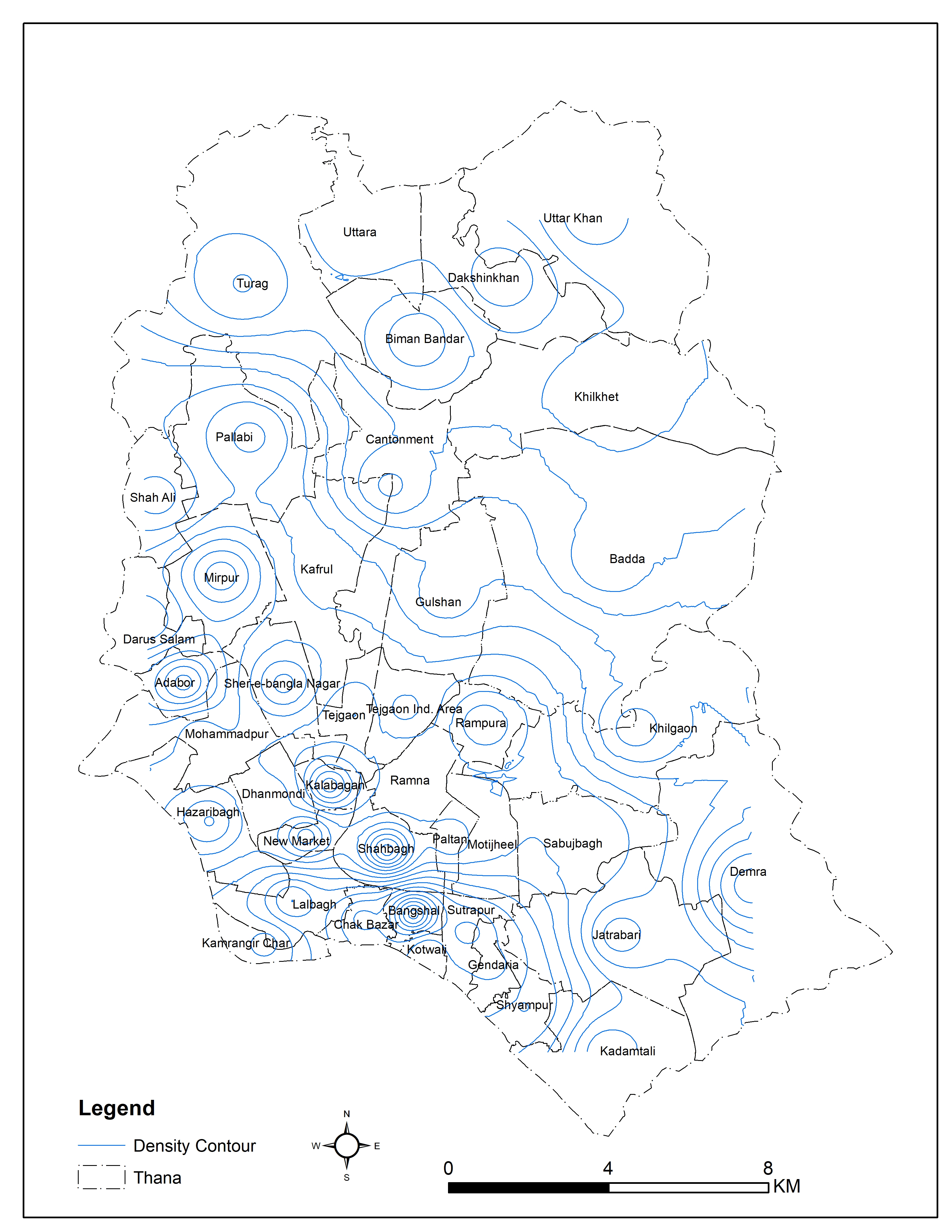

Supplement: S2 Fig — a This population contour map was developed using the methods explained in H. Khatun, N. Falgunee, M. J. R. Kutub (2015), Analyzing urban population density gradient of Dhaka Metropolitan Area using Geographic Information Systems (GIS) and Census Data, GEOGRAFIA OnlineTM Malaysian Journal of Society and Space 11 issue 13 (1–13). (TIFF) [file pone.0241437.s003.tiff]
